# Plasma miRNA as Biomarkers for Assessment of Total-Body Radiation Exposure Dosimetry

**DOI:** 10.1371/journal.pone.0022988

**Published:** 2011-08-17

**Authors:** Wanchang Cui, Jinfang Ma, Yulei Wang, Shyam Biswal

**Affiliations:** 1 Department of Environmental Health Sciences, Bloomberg School of Public Health, Johns Hopkins University, Baltimore, Maryland, United States of America; 2 Genomic Assays R&D, Life Technologies, Foster City, California, United States of America; Mizoram University, India

## Abstract

The risk of radiation exposure, due to accidental or malicious release of ionizing radiation, is a major public health concern. Biomarkers that can rapidly identify severely-irradiated individuals requiring prompt medical treatment in mass-casualty incidents are urgently needed. Stable blood or plasma-based biomarkers are attractive because of the ease for sample collection. We tested the hypothesis that plasma miRNA expression profiles can accurately reflect prior radiation exposure. We demonstrated using a murine model that plasma miRNA expression signatures could distinguish mice that received total body irradiation doses of 0.5 Gy, 2 Gy, and 10 Gy (at 6 h or 24 h post radiation) with accuracy, sensitivity, and specificity of above 90%. Taken together, these data demonstrate that plasma miRNA profiles can be highly predictive of different levels of radiation exposure. Thus, plasma-based biomarkers can be used to assess radiation exposure after mass-casualty incidents, and it may provide a valuable tool in developing and implementing effective countermeasures.

## Introduction

In the event of an improvised nuclear device attack or disaster in a nuclear power plant, hundreds of thousands of people may be exposed to various doses of radiation. Exposure to ionizing radiation is associated with induction of acute radiation syndrome (ARS), also known as radiation sickness, which is characterized by nausea, vomiting, diarrhea, skin damage, and can include loss of bone marrow, internal bleeding, and death. Depending on the exposure dose, the symptoms of ARS can appear within hours to weeks. The categories relevant to radiation exposure in populations have been defined [1]. Subjects receiving doses less than 2 Gy are asymptomatic or have mild symptoms; subjects receiving doses from 2 to 6 Gy need immediate care, with variable survival prognoses; and radiation doses >6 Gy will likely be lethal. At doses >2 Gy, the hematopoietic system syndrome appears in weeks to 2 months and mortality can occur within 1 month; at doses of 8 Gy to 12 Gy, gastrointestinal effects occur within a week after the exposure and death can occur within 10 days; at doses >15 Gy, the central nervous system will be affected and fatalities can occur within 2 days [2].

It is a challenge for medical responders to triage individuals who are minimally exposed and do not need treatment compared to those who received high dose radiation and need immediate treatment [3]. The current strategy to assess individual radiation exposure is based on symptoms developed over time. For example, individual radiation dose is assessed by determining the time to onset and severity of nausea and vomiting, lymphocyte depletion kinetics, and cytogenetic analysis [1]. These methods are very time-consuming and difficult to apply to an exposed population in a real time scenario. In addition, the latent period (from radiation exposure to symptom onset) is a critical time for therapeutic intervention because many effective treatments such as antibiotics or hematopoietic cytokines need to be administered before radiation symptoms appear. Therefore, there is an urgent need to develop biochemical or molecular biomarkers that can help in implementing effective countermeasures of radiation injury to affected individuals and provide basis for treatment decisions. The levels of amylase, C-reactive protein, and other proteins have been studied as radiation biomarkers [4]. However, the action of these biomarkers can be affected by physiological conditions such as inflammation or bacterial infection. γ-H2AX foci at DNA double-strand break sites in peripheral blood lymphocytes are also being developed as biomarkers for determining the extent of radiation exposure [5].

MicroRNAs (miRNAs) are small (typically about 22 nucleotide in size) regulatory RNA molecules that modulate the activity of specific mRNA targets and play an important role in many biological processes. Circulating miRNAs (in plasma, serum or other bodily fluids) are being assessed as biomarkers for various pathological and physiological conditions. For example, circulating miRNAs are being investigated as stable, blood-based biomarkers for prostate cancer, pancreatic cancer, acetaminophen-induced liver damage, and pregnancy [6], [7], [8]. There are many advantages to using circulating miRNAs as biomarkers as miRNA expression is frequently altered in diseases or during organ damage, and many miRNAs are tissue specific, stable, and can be used in a high-throughput analysis with ease. High-throughput miRNA expression analysis is extremely sensitive, rapid and flexible PCR based assay and thus requires less effort compared to traditional methods. The limitation of using plasma or serum samples for miRNA analysis is that no specific miRNA is available to use as an endogenous control to normalize the relative quantity of miRNA in the plasma or serum. In contrast, house keeping genes such as β-actin and GAPDH are commonly used in quantitative RT-PCR analysis studying gene expression in various tissues. Michell et al (2008) used synthetic *Candida elegans* miRNAs as spiked-in controls [7]. Although this method is helpful to normalize the variability in plasma or serum RNA extraction, it cannot be used to normalize the variability in biological samples. Using a murine model of single exposure total body irradiation (TBI), we investigated whether circulating miRNA in plasma can differentiate exposure to radiation doses of 2 Gy and higher and developed a normalization strategy to analyze the data.

## Materials and Methods

### Animals and animal irradiation

All animal experimental protocols were in agreement with the guidelines established by the institute and approved by the Institutional Review Board at the Johns Hopkins University Animal Care and Use Committee(Protocol No. MO08H274). C57BL6 mice (male, 6–8 weeks old) were exposed to different doses of gamma radiation (Total Body Irradiation, TBI) using a GammaCell 40 irradiator with a Cesium-137 source (Atomic Energy of Canada Limited) at a dose rate of 52 cGy/min. Control mice were sham-exposed.

### Blood collection and plasma preparation

Peripheral blood was collected after radiation by heart puncture at 6 h or 24 h using BD Vacutainer K2 EDTA tubes (BD Biosciences 367841). After collection of whole blood in BD Vacutainer K2 EDTA tubes, these tubes were immediately and gently inverted for 8–10 times. Plasma was processed within two hours after blood collection. To harvest the cell-free plasma, we used a two-step centrifugation method [9]. Blood samples were first centrifuged at 2000 rpm for 10 min at room temperature (according to BD Vacutainer instructions). The supernatant was centrifuged again at 16000 g for 10 min at 4°C to remove residual blood cells. Plasma samples were stored at −80°C. To test whether there was residual platelet contamination in our plasma samples, we quantified platelet counts using HemaVet ® HV950FS Multispecies Hematology Instrument (Drew Scientific, Inc). Because platelet miRNAs from residual platelets or from platelet lysis would show up in the plasma miRNA array, we compared the platelet specific gene Itga2b in both plasma and whole blood. 10% of plasma and 10% of buffy coat from the same blood sample were used for RNA extraction. The ratio of Igta2b between 10% of plasma and 10% buffy coat represents the ratio of Itga2b between plasma and whole blood.

### RNA extraction

RNA was extracted using mirVana PARIS kit (Ambion AM1556). Because there is no suitable endogenous control for plasma RNA, miR159a, a miRNA from *Arabidopsis thaliana* not present in mammalian tissues, was added as a spiked-in control. Mouse plasma (200 µl) was added to 205 µl 2× denaturing solution and mixed thoroughly. Synthetic miR159a (IDT, City State) was then added to the mixture and mixed thoroughly. After chloroform addition and phase separation, the aqueous layer was mixed with 1.25 volumes of absolute ethanol. Then, the solution was loaded onto the cartridge provided with the mirVana miRNA isolation kit. The columns were washed according to the manufacturer's instructions, and the RNA was eluted in 50 µl nuclease free H_2_O (95°C). To minimize DNA contamination, the eluted RNA was treated with DNase I (Ambion AM1906).

### microRNA profiling

Three microliters of total RNA was reverse transcribed using the Megaplex™ RT Primers Rodent Pool A and Pool B (Life Technologies, Foster City, CA), enabling miRNA specific cDNA synthesis. The resulting cDNAs were pre-amplified for 12 cycles using the corresponding Megaplex™ PreAmp Primers Rodent Pool A or Pool B and TaqMan PreAmp Master Mix according to the manufacturer's protocol (Life Technologies, Foster City, CA). The pre-amplified cDNAs were diluted 4 fold with 0.1×TE (pH 8.0). miRNA expression profiling was performed using Taqman Rodent MicroRNA Array Card A and Card B (Applied Biosystems) containing all 518 mature mouse miRNAs in miRBase 10.1 (http://microrna.sanger.ac.uk). PCR amplification and signal detection was performed using the Applied Biosystems 7900HT Fast RT-PCR System.

### miRNA expression analysis

The miRNA expression results were analyzed using Real-Time StatMiner software (Integromics, Madrid, Spain). miRNAs were first normalized using global mean normalization across all miRNA TaqMan arrays to achieve the same mean Ct (determined using only miRNAs passing detection threshold (Ct<32) for each array). To minimize potential noise introduced by measurements below detection threshold, miRNAs with Ct>35 in all groups were filtered out. miRNAs that were differentially expressed for each of the three radiation doses after 6 hr and 24 hr were identified using a LIMMA modified *t*-test (p<0.05). Hierarchical clustering (Pearson's Dissimilarity algorithm) was performed using Partek Genomic Suite (Partek Inc., St Louis, MO, USA). Average-linkage hierarchical clustering analysis and visualization was performed using the CLUSTER and TREEVIEW programs, respectively, (software available at http://genome-www5.stanford.edu/resources/restech.shtml).

### Construction and validation of miRNA-based prediction models for radiation doses

A 40-sample training set containing 10 samples for each radiation dose collected from mice (n = 10/group) exposed to 0, 0.5, 2.0, and 10 Gy at 6 h and 24 h was used to select classifier miRNAs and construct a prediction model. To establish the prediction models for radiation doses, Ct values of a low abundance miRNA with Ct values equal or above 32 in all groups were replaced with a Ct value of 32 to perform stricter statistical analysis. miRNA-based prediction models for radiation doses were constructed and validated by prediction analysis of microarrays, using a statistical package (http://www-stat.stanford.edu/~tibs/PAM/) that applies nearest shrunken centroid analysis and cross-validation to determine the minimal set of predictor genes that achieve optimal prediction accuracy for sample classification [10]. The optimal number of classifier genes was determined by choosing an optimal threshold with 0% training error. For validation of the prediction models, a 10-fold cross validation was performed on the training set. The 40 training samples were partitioned into 10 bins, with equal representation of radiation doses similar to the initial set of samples. Nine bins were used for standardization purposes to construct the prediction models, and the radiation dose of samples belonging to the remaining bin was predicted. Classification errors were calculated for each dose to evaluate the performance of each prediction model.

## Results

### Survival of mice after exposure to different doses of radiation

**Figure 1:**
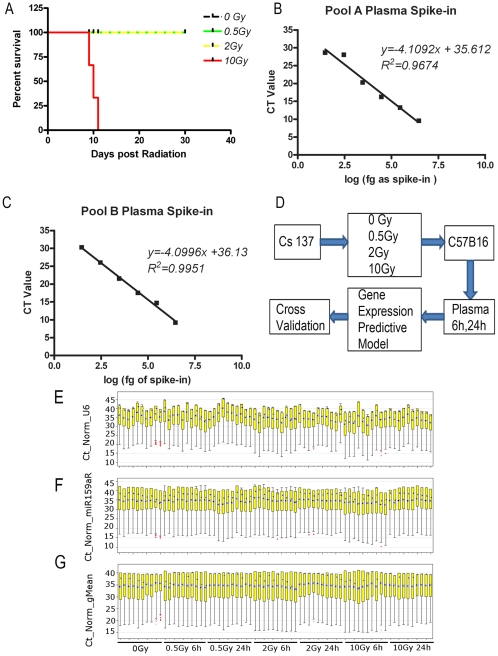
Experimental design and scheme for development of radiation response expression signature. **A:** C57BL6 mice survival after TBI. C57BL6 mice (male, 6–8 weeks old) were irradiated with 0 Gy, 0.5 Gy, 2 Gy and 10 Gy TBI. Animal survival was monitored for 30 days (N = 10 in each group). **B & C:** The linear range of spiked-in miRNA control. miR159a from 0.03 pg to 3000 pg (in 10 fold increase) showed a linear reaction in both Array A and Array B reactions. X axis: log of spiked-in miRNA control (in picogram); Y axis: Ct values detected using real time PCR. **D:** Experimental design for development of radiation response signature. Plasma was collected from 0 (control), 0.5-, 2-, and 10 Gy-irradiated C57BL6 mice at 6 h and 24 h after exposure. miRNA array analyses were performed on the RNA isolated from n = 10 replicates from each condition, and metagene profiles were developed to represent 4 different levels of radiation exposure at 2 time points. For the metagene profiles, a 10-fold cross validation was performed, which revealed the highly predictive nature of these metagene profiles. **E–G:** showed the box plot distribution of normalized Ct values in each sample using three different normalization methods. **E:** Ct distribution after normalization using mammalian U6 as the normalizer; **F:** Ct distribution after normalization using spiked-in miR159a as the normalizer; **G:** Ct distribution after normalization using the global mean as the normalizer. The yellow boxes represent the first quartile (Q1) and third quartile (Q3) of Ct values in each sample, while the whisker bars represent the upper and lower adjacent values of Cts in each sample. Blue triangles represent median Ct values and black triangles represent mean Ct values.

First, we studied the survival of mice receiving 0.5, 2, and 10 Gy TBI. C57BL6 mice (6–8 weeks old, male, n = 10 per group) were exposed to a single dose of 0.5, 2, or 10 Gy TBI from a Cs137 gamma source at a dose rate of 52 cGy/min. The survival results are shown in Figure 1A. We confirmed that 10 Gy TBI was lethal and resulted in 100% mortality within 2 weeks; while there was no mortality at a dose of 2 Gy or lower in 30 days.

### Establishment of spiked-in controls

We established the linear range of spike-in miRNA in mouse plasma. Different quantities of miR159a were spiked into 200 µl plasma of untreated C57BL6 mice. RNA from the plasma was extracted and reverse transcribed, and RT-PCR was performed to detect the spiked-in miRNA levels. As shown in Figure 1B&C, spiked miR159a in the range of 3000 pg to 0.03 pg (serial 10-fold dilutions) was detected in the linear range in both Pool A and B of the Taqman assay. The yield from this titration produced an excellent linear regression correlation (R2 = 0.967 and R2 = 0.995, respectively) suggesting that miR159a can function as an optimal spiked-in control. For later experiments, we chose to spike-in 30 pg of miR159a to 200 µl of plasma sample.

### Experimental design

The experimental design is shown in Figure 1D. To study the plasma miRNA expression alteration due to different radiation doses, C57BL6 mice were exposed to the same three doses of TBI as shown in Figure 1A. These three doses were chosen because each dose represents an exposure with different pathophysiological implications. Blood was drawn at 6 h and 24 h after radiation and plasma was prepared and stored at −80°C. Plasma miRNA was extracted and used for miRNA Taqman array. Microarray analyses were performed as described in the Materials and Methods.

### Normalization strategies

Current approaches to normalize the circulating miRNAs include the use of established endogenous miRNAs, spiked-in miRNAs or global mean of miRNAs. To select the best method to normalize the miRNA expression level, we tested normalization based on mammalian U6, spiked-in miR159a, or global mean of miRNAs with Ct<32. First, we normalized the expression value using mammalian U6, which is widely used as an endogenous control for tissue or cell miRNA normalization. As shown in Figure 1E, miRNA expression levels normalized using mammalian U6 showed high variation within individual treatment groups. Normalization based on spiked-in miR159a showed less variation within each group compared to the normalization based on U6 (Figure 1F). Normalization based on the global mean of each plate showed even better results within each group compared to normalization based on miR159a as a normalizer (Figure 1G). Since the global mean expression value is the best ranked normalization factor, which significantly reduces technical variation, we chose to normalize the expression values based on global mean of miRNAs.

### Contamination from platelets

Because platelets are the most easily lysed cells during plasma preparation and it has been shown that platelets contain miRNAs [11], there is a possibility of platelet miRNA contamination of plasma miRNAs. Platelets have been shown to be extremely resistant to radiation. Radiation doses as high as 75000 rads (which equals to 750 Gy) caused no damage to platelets [12]. Platelet miRNA might contaminate plasma miRNA in two ways. Firstly, there might be residual platelets in the plasma after centrifugation. Secondly, platelets might be lysed during the preparation of plasma and release platelets miRNAs into the plasma. To establish whether there was platelet contamination, and if there was, whether the contamination was similar in different samples, we did three experiments. Firstly, we quantified the residual platelets in plasma after centrifugation. As shown in Figure S1B, there were 583.4±44.4 k/µl platelets in the whole blood. After first centrifugation, there were 13.0±2.3 k/µl platelets remaining in the plasma. After the second centrifugation, there were only 4.8±0.3 k/ul platelets remaining in the plasma. Therefore, we removed 97.8% platelets after first centrifugation and 99.2% platelets after second centrifugation. Because platelet counts after second centrifugation was in the lowest linear range of HemaVet machine we used (the linear range is 1 to 4000 k/µl), we believe that the removal of platelets from plasma was very efficient. Secondly, we did realtime PCR to measure the platelet specific gene product Itga2b (also called glycoprotein IIb, GPIIb) in the whole blood and plasma. Realtime PCR result showed that Itga2b expression was dramatically decreased in the plasma compared to the whole blood. The arbitrary Ct values of whole blood Igta2b was 16±0.45, the arbitrary Ct values of plasma Igta2b after first centrifuge was 32±0.21 and the arbitrary Ct values of plasma igta2b after second centrifuge was 36±0.53 (based on the line draw). Based on the delta delta CT method to calculate the fold change in gene expression in plasma and whole blood, the fold decrease from whole blood to plasma after first centrifuge was 55521 and to plasma after second centrifuge was decreased to 1454803 folds (as shown in Figure S1A and S1C). This also suggests that even if there were platelet miRNA contaminations, the level of contamination was very low. The Ct values of Itga2b in plasma samples had a normal distribution, suggesting that even if there were platelet lysis during plasma preparation, the contamination would not vary much in different samples. Thirdly, we compared the most abundant miRNAs from platelets and plasma. Since plasma miRNA levels are very low, tiny contamination from platelet might affect the constituent of plasma miRNA. We studied the distribution of the 10 most abundant platelet miRNAs in plasma and the distribution of the 10 most abundant plasma miRNAs in platelets. Because there is no miRNA data from mouse platelets and miRNAs are known to be much conserved between species, we believe it is acceptable to use the miRNA data from human platelets to compare with mouse samples. We used the human platelet miRNAs abundance list from Landry P et al [11]. We showed the comparison in Figure S1D and S1E. We showed that the 10 most abundant platelet miRNAs ranked from the 13th most abundant to 227th most abundant in plasma miRNAs (Figure S1D). The 10 most abundant plasma miRNAs ranked from 12th to 150th in the platelet, and three of the most abundant plasma miRNAs were absent from the platelet list (Figure S1E). Our results suggest that there were negligible platelet miRNA contaminations of our plasma samples.

### Differential expression of plasma miRNA after ionizing radiation

**Figure 2:**
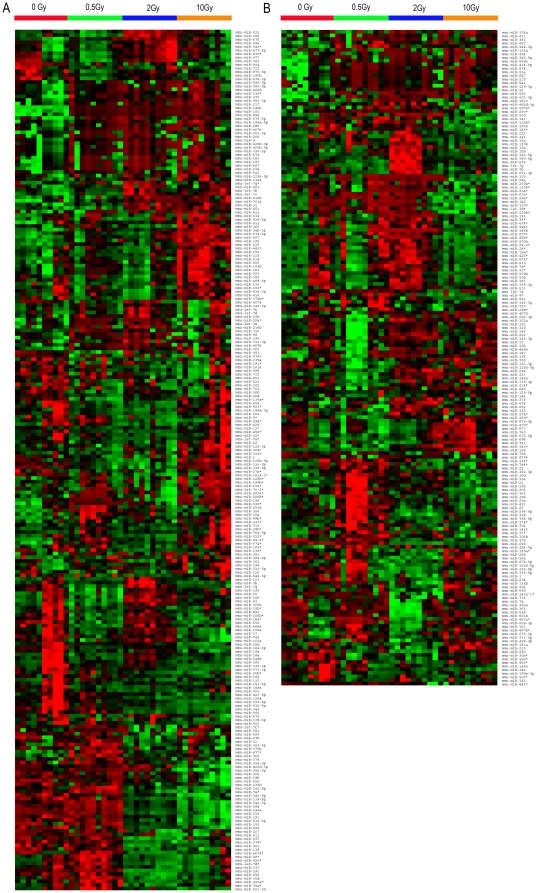
Different doses of ionizing radiation affect plasma miRNA expression profiles. **A:** Hierarchical clustering of 40 samples (10 controls and 10 samples from each of the following radiation doses 0.5 Gy, 2 Gy and 10 Gy at 6 h) using 239 differentially expressed miRNAs (p<0.05). **B:** Hierarchical clustering of 40 samples (10 controls and 10 samples from each of the following radiation doses 0.5 Gy, 2 Gy and 10 Gy at 24 h) using 182 differentially expressed miRNAs (p<0.05). The level of expression of each gene in each sample is represented using a red-black-green color scale as shown in the key (high expression is depicted as red, and low expression is depicted as green, unchanged expression is depicted as black). The radiation level of each sample is color coded as illustrated.

To identify a miRNA expression signature in the plasma in response to ionizing radiation, miRNA profiles of plasma samples collected from 40 mice exposed to four radiation doses (0, 0.5, 2.0, and 10 Gy, n = 10/dose) were analyzed using TaqMan Rodent MicroRNA Array Card A and Card B containing 518 mature mouse miRNAs. A set of 239-miRNA and 182-miRNA signature was determined at 6 h and 24 h post radiation exposure, respectively. As shown in Figure 2 A&B, hierarchical clustering analysis using these miRNA signatures revealed distinct patterns of miRNA expression and clearly distinguished the non-irradiated samples from the irradiated samples. Furthermore, it is evident that clusters of miRNAs could discretely separate the samples as a function of radiation dose. These results suggest that there was alteration in the expression of a specific set of miRNAs in mice blood after exposure to various doses of radiation.

### Dose-dependent differential expression of miRNA

**Figure 3:**
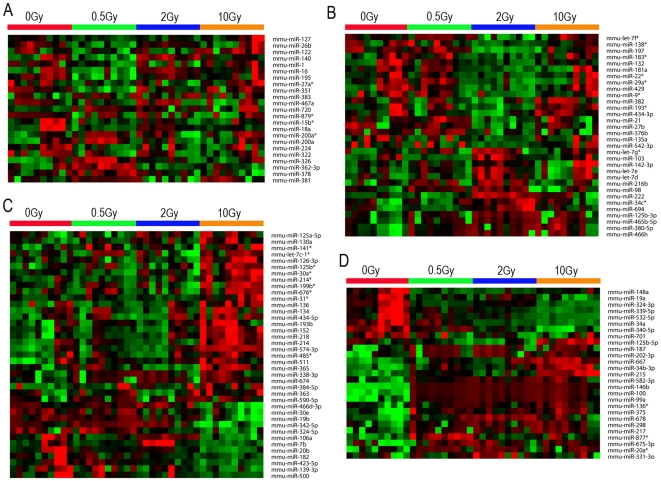
Differential expression of plasma miRNA after different radiation doses at 6 h. The heatmap depicts the expression pattern of miRNAs showing significantly differential expression compared to control group. miRNAs that were significantly changed compared to the control group and uniquely expressed in 0.5 Gy,2 Gy and 10 Gy radiation dose were shown in **A** to **C**. **A:** 23 miRNAs that were uniquely changed in the 0.5 Gy 6 h group compared to the control group. **B:** 32 miRNAs that were uniquely changed in the 2 Gy 6 h group compared to the control group. **C:** 39 miRNAs that were uniquely changed in the 10 Gy 6 h group compared to the control group. **D:** 27 miRNAs that were significantly changed in all three irradiation groups compared to the control group. High expression is depicted as red, and low expression is depicted as green, unchanged expression is depicted as black.

**Figure 4:**
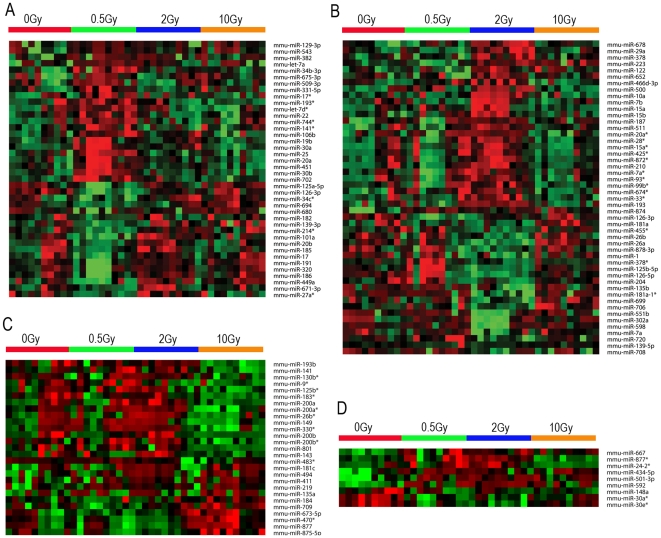
Differential expression of plasma miRNA after different radiation doses at 24 h. The heatmap depicts the expression pattern of miRNAs showing significantly differential expression compared to the control group. miRNAs that were significantly changed compared to the control group and uniquely expressed in one radiation dose are shown in **A** to **C**. **A:** 40 miRNAs that were uniquely changed in the 0.5 Gy 24 h group compared to the control group. **B:** 49 miRNAs that were uniquely changed in the 2 Gy 24 h group compared to the control group. **C:**27 miRNAs that were uniquely changed in the 10 Gy 24 h group compared to the control group. **D:** 9 miRNAs that were significantly changed in all three irradiation groups compared to the control group. High expression is depicted as red, and low expression is depicted as green, black means no change.

Given the pattern of plasma miRNA expression after exposure to radiation, we identified the miRNAs that have a distinct expression at specific doses and time points (clustered using Pearson's dissimilarity as distance measure). There were 23, 32, and 39 unique miRNAs (metagenes) that significantly changed after exposure to 0.5, 2, and 10 Gy ionizing radiation at 6 h, respectively (Figure 3A–C). We also analyzed miRNAs that were differentially expressed in response to different doses of radiation at 24 h. There were 40, 49, and 27 unique miRNAs that were significantly altered after exposure to 0.5, 2, and 10 Gy ionizing radiation at 24 h, respectively (Figure 4A–C). As shown in Figure 3D, there were 35 miRNAs that were significantly changed after exposure to all three doses of radiation (0.5, 2 and 10 Gy) at 6 h compared to unirradiated samples. Nine miRNAs were significantly changed after exposure to all three doses of radiation (0.5, 2 and 10 Gy) at 24 h compared to unirradiated samples (Figure 4D). We found that the differential expression of miRNAs after radiation exposure was specific to radiation doses. These data suggest that radiation exposure induces dose-dependent changes in plasma miRNA expression.

### Temporal expression of plasma miRNA after radiation

**Figure 5:**
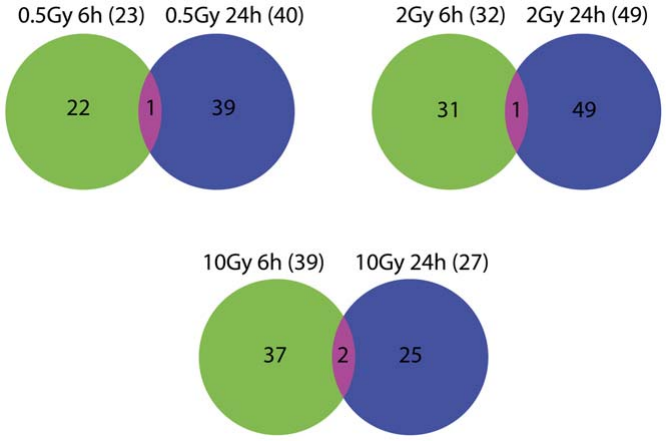
Venn diagram showing the time-dependent expression of miRNAs. Significantly changed miRNAs at the same dose but different time points were used for drawing the Venn diagram. The miRNA lists were from Figure 3 **A** to **C** and Figure 4 **A** to **C**. miRNAs that were uniquely changed at 6 h were colored in lime green. miRNAs that were uniquely changed at 24 h were colored in blue. miRNAs that were changed at both 6 h and 24 h were colored in red.

We found a temporal relationship between the expression of miRNAs and radiation by comparing the miRNAs from the same dose to different time points (Figure 3&4). As shown in Figure 5, there were 23 and 40 miRNAs that were uniquely differentially expressed at 0.5 Gy for 6 h and 24 h, respectively. Among them, only one was common at both time points. Similarly, there were 32 and 49 miRNAs that were uniquely differentially expressed at 2 Gy for 6 h and 24 h, respectively. Of these, only 1 miRNA was differentially expressed at both time points. There were 39 and 27 miRNAs that were uniquely and differentially expressed at 10 Gy for 6 h and 24 h, respectively. Of these, only 2 miRNA were differentially expressed at both time points. This suggests that the expression of plasma miRNA after exposure to radiation is a dynamic process.

### Development of radiation response signature that can predict radiation doses

**Figure 6:**
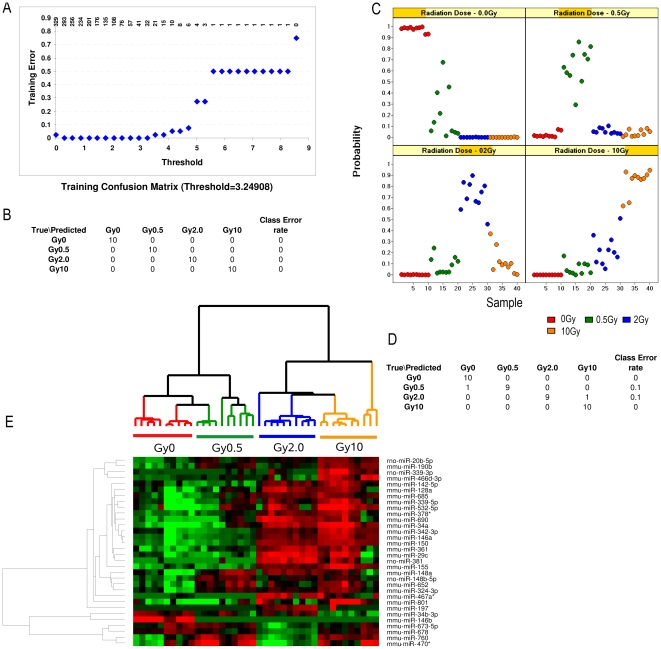
miRNA expression profiles that distinguish different levels of radiation exposure at 6 h. **A:** Determination of the optimal set of 32 predictor miRNAs and threshold with minimal (0%) training error. **B:** The confusion matrix table showed the individual training error for each class. **C:** 10-fold cross validation of the 32-predictor model on the training set (see detailed description in Methods). Probability of being predicted as normal or each individual radiation dose was plotted for each sample. The radiation level of each sample is color coded as illustrated (red, 0 Gy; green, 0.5 Gy; blue, 2 Gy; orange, 10 Gy). **D:** The confusion matrix table showed the individual cross validation error for each class. **E:**hierarchical clustering of the 32-miRNA in prediction model for radiation doses at 6 h. High expression is depicted as red, and low expression is depicted as green, unchanged expression is depicted as black.

Since we were able to develop a miRNA signature reflective of radiation dosage, we attempted to find the expression signatures or metagenes that could accurately predict the exposure to various doses of radiation. We used the PAM statistical package to determine the minimal sets of miRNAs that can predict specific radiation doses at 6 h and 24 h. As shown in Figure 6A, while training the 6 h time point samples, a 32 miRNA model was identified; and its training error was 0% (Figure 6B). To cross validate the model, we used a 10-fold cross validation. As shown in Figure 6C, the 32 miRNA metagene can distinguish all radiation doses in comparison to the unirradiated mice. The predictive error was 0% for the unirradiated and 10 Gy 6 h comparison and 10% for the 0.5 Gy 6 h and 2 Gy 6 h groups. A heatmap of these 32 miRNAs is shown in Figure 6E. The overall accuracy, sensitivity, and specificity of the 32 miRNA signature for the 6 h group are shown in Table 1. The 32 miRNA signature achieved 97.5% overall accuracy in the 6 h groups; 100% sensitivity in the unirradiated and 10 Gy groups; 90% sensitivity in the 0.5 Gy and 2 Gy groups; and 97% specificity in the unirradiated and 10 Gy groups; and 100% specificity in the 0.5 Gy and 2 Gy groups.

**Table 1:** Prediction of radiation doses with PAM using the 32-miRNA expression signature and the 12-miRNA expression signature for 6 h and 24 h, respectively.

Treatment	Overall Accuracy^a^(%)	Sensitivity^b^(%)	Specificity^c^(%)
6 h Post TBI			
0 Gy	97.5	100	97.0
0.5 Gy 6 h	97.5	90.0	100
2 Gy 6 h	97.5	90.0	100
10 Gy 6 h	97.5	100	97.0
24 h Post TBI			
0 Gy	97.5	100	97.0
0.5 Gy 24 h	97.5	90.0	100
2 Gy 24 h	100	100	100
10 Gy 24 h	100	100	100

PAM: statistical package, Prediction Analysis of Microarrays.

a Prediction accuracy was determined by 10-fold cross-validation on the 40 samples receiving different doses of radiation. Accuracy = (the number of samples predicted correctly)/(total number of samples analyzed).

b Sensitivity = (the number of positive samples predicted)/(the number of true positives).

c Specificity = (the number of negative samples predicted)/(the number of true negatives).

**Figure 7:**
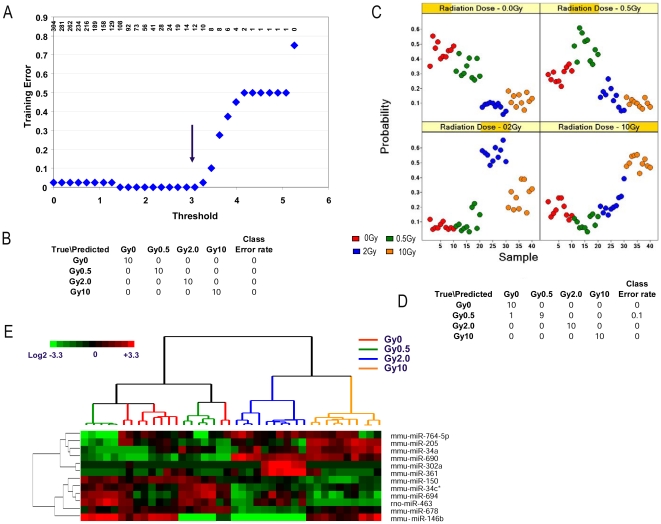
miRNA expression profiles that distinguish different levels of radiation exposure at 24 h. **A:** Determination of the optimal set of 12 predictor miRNAs and threshold with minimal (0%) training error; **B:** The confusion matrix table showed the individual training error for each class; **C:** 10-fold cross validation of the 12-predictor model on the training set (see detailed description in Methods). Probability of being predicted as normal or each individual radiation dose was plotted for each sample. The actual radiation level of each sample is color coded as illustrated (red, 0 Gy; green, 0.5 Gy; blue, 2 Gy; orange, 10 Gy). **D:** The confusion matrix table showed the individual cross validation error for each class. **E:** Hierarchical clustering of the 12-miRNA in prediction model for radiation doses at 24 hr. High expression is depicted as red, low expression is depicted as green, and unchanged expression is depicted as black.

In addition, we performed the training on the 24 h samples and found a 12 miRNA model that could distinguish all four radiation doses with 0% training error (Figure 7A&B). As shown in Figure 7C, the 12 miRNA model could distinguish 0, 0.5, 2, and 10 Gy. The predictive error was 10% for the 0 Gy and 0.5 Gy group at 24 h and 0% for all other groups. Figure 6E shows the heatmap of the 12 miRNAs that could distinguish the specific radiation doses at 24 h. As shown in Table 1, at 24 h, the 12 miRNA signature achieved 97.5% overall accuracy in the unirradiated and 0.5 Gy groups; 100% overall accuracy in the 2 and 10 Gy group comparison; 100% sensitivity in the unirradiated group and 2 and 10 Gy groups; 90% sensitivity in the 0.5 Gy group; 97% specificity in the unirradiated group; and 100% specificity in the 0.5, 2 and 10 Gy groups.

## Discussion

In the case of a nuclear disaster or nuclear device attack, a triage diagnostic screening approach will be very useful to rapidly and accurately determine the severely radiation-exposed individuals from those who were exposed to sublethal dose (<2 Gy). Molecular biodosimetry tools can be extremely valuable assets for medical triage and patient management and implementation of effective countermeasures. Many studies have demonstrated that after radiation exposure, interventions aimed at ameliorating the effects of radiation must be applied early. Delayed administration of countermeasures is not effective. Because many symptoms of ARS are delayed, it is important to use predictive biomarkers that can distinguish the level of radiation exposure. Traditional methods such as lymphocyte depletion or cytogenetic analysis [1] are very time consuming and labor intensive. Gene-expression or molecular biomarkers are being tested to detect radiation exposure for prompt and effective implementation of countermeasures.

We chose the three radiation doses (0.5 Gy, 2 Gy and 10 Gy) because each radiation dose has its own medical significance. It is well established that 0.5 Gy exposure causes no acute health effects, whereas 2 Gy is myelosuppressive and immunosuppressive and 10 Gy is in upper range of survival with supportive care and cytokines. In a scenario following a large nuclear event, it is critical to identify those individuals who will benefit from medical intervention at an early stage. But it is also crucial to exclude and reassure patients who do not need medical intervention since the medical resources will be extremely limited. People receiving radiation doses from 2 Gy to 10 Gy need immediate medical attention. The best estimate for the LD50/50 in humans is in the 3.5 to 4.5 Gy range, however, this value can be roughly doubled through the use of antibiotics, platelet and cytokine treatment [13]. In the dose of approximately 7–10 Gy, bone-marrow transplantation is a useful option (above 10 Gy is usually lethal because of lethal gastrointestinal damage) [14]. Therefore, we believe a biomarker to distinguish 2 Gy and 10 Gy is utmost important because people receiving these doses all need medical attention. In fact, researchers have used these three radiation doses for biomarker studies [15], [16], [17]. Further studies using other radiation doses such as 4 Gy or 6 Gy will give us a better understanding of the plasma miRNAs after radiation exposure.

The type of anticoagulant used in plasma collection tubes is very important to consider. Spray-dried EDTA is the preferred anticoagulant for quantitative molecular diagnostic tests. Collecting plasma using heparin will interfere with PCR reaction because heparin will bind to the calcium and magnesium present in the master mix for PCR reactions; citrate will dilute plasma and can cause hemolysis( http://www.bd.com/vacutainer/faqs/). To get cell-free plasma, we used a two-step centrifugation method, which has been shown to be effective in producing cell-free plasma [9]. Platelets are stable in EDTA-anticoagulated blood for 24 hrs [18]. Our blood samples were processed within 2 hours after blood draw. Also we showed that there was minimal platelet lysis contamination by measuring the Itga2b levels. At least 99.2% of platelets were removed from plasma after the second centrifugation. Furthermore, we showed that there was almost no platelet specific gene itga2b in the plasma after the second centrifugation. The expression level of itga2b was around 1.5×106 fold lower than that in the whole blood. The most convincing results are from the comparison of the most abundant miRNAs from platelets and plasma. If there was significant miRNA contamination from platelets, the 10 most abundant platelet miRNAs should rank very high and closely in plasma samples. Also if there was significant contamination from platelets, the 10 most abundant plasma miRNAs should rank very high in the platelet list. The fact that the first three most abundant plasma miRNAs were not detected in the platelet samples suggests that platelets are not a major source of plasma miRNAs. In summary, our results suggest that there were negligible platelet miRNA contaminations of our plasma samples.

There is a strong rationale to develop non-invasive biomarkers reflective of radiation exposure based on molecular changes such as DNA, RNA and plasma protein [17], [19], [20]. The expression of mRNA isolated from mononuclear cells has been used to predict radiation doses; however, mRNA is intrinsically unstable [21]. A protein-based approach provides an alternative and complementary approach to microarray technology for the identification and validation of proteins. However, there are concerns associated with the protein-based approach such as complexity of protein compositions, posttranslational modifications, low abundance of proteins of interest, and difficulty in developing detection methods with high affinity [22], [23]. Plasma B1 DNA seems to be a simple and accurate biomarker for detecting radiation exposure in biological systems [20]; however, it is known that plasma DNA levels can increase as a result of many illnesses [24], [25], [26]. Unlike mRNA-based biomarkers, miRNA based biomarkers are relatively stable and their total number is small. The measurement of miRNAs in plasma has the advantage of reduced noise compared to measurements of mRNA from blood cells, which can have variation due to differences in the cell number due to infection and other secondary conditions unrelated to exposure. However, one advantage to using mRNA as a biomarker instead of miRNA is the presence of well-established internal controls in mRNA samples.

The lack of endogenous controls is a challenge to normalize the miRNA data. We have demonstrated that global normalization across the samples is the optimal choice. Our data show that gamma radiation causes quantifiable and reproducible miRNA gene expression changes in plasma in a time- and dose-dependent manner. miRNA expression profile can predict and distinguish the level of radiation exposure in mice, ranging from 0.5 Gy to 10 Gy. The 32 miRNA metagene profile at 6 h demonstrated 97.5% accuracy in distinguishing non-irradiated mice from those exposed to 0.5, 2, or 10 Gy. The 12 miRNA metagene profile at 24 h post radiation demonstrated at least 97.5% accuracy in distinguishing non-irradiated mice from those exposed to 0.5, 2, or 10 Gy. Combined with other discrimination indices of radiation exposure, we showed that this miRNA profiling method can help to effectively predict radiation casualty incidents.

We found about 50% of all the known miRNAs in the plasma. The high abundance of many circulating miRNAs in the plasma suggests they might have potential biological roles. Among the miRNAs in the plasma, many have been shown to have differential expression in cells or tissue after ionizing radiation, such as miR-142-5p [27], miR-339, hsa-miR-342, hsa-miR146a, hsa-miR-29c, hsa-miR155, hsa-miR-197, hsa-miR-34b [28], and miR-29c [29]. We also found that miR34a was elevated in all plasma samples exposed to radiation. miR34a has been shown to be up-regulated after ionizing radiation both in vitro and in vivo [30]. After exposure to gamma radiation, p53 is activated through ATM-kinase and transactivates the expression of different members of the miR34 family through consensus binding sites. miR34 genes are then processed by DROSHA and DICER complexes. The mature miR-34 incorporates in the RISC complex and mediates the inhibition of translation or RNA degradation of their targets, such as Bcl-2 and Cyclin D1 [31]. The activation of p53 increases the levels of the miR34 family, which are direct targets of p53. The activation of the miR34 family then regulates their target proteins such as CDK4 and Rb to regulate the cell cycle. Circulating miRNAs might act as extracellular messengers mediating short- and long-range cell-cell communication similar to roles played by small RNAs in C. elegans and plants [32], [33]. Further studies are needed to explore the potential biological roles of circulating miRNAs.

Radiation at doses from 2 Gy to 10 Gy causes damage mainly to the bone marrow and GI tract. We expect that these two radiosenstive organs might release miRNAs to the plasma after radiation exposure. Currently there are limited studies about the tissue specific or tissue enriched miRNAs. miR125a and miR125b have been shown to be enriched in bone marrow [34] and there are about 30 miRNAs shown to be enriched in the small intestine compared to liver [35]. Among them, only miR-142-5p showed up in our 6 h metagene and none showed up in our 24 h metagene.

We studied the tissue distribution of the 32 and 12 miRNAs we found in the 6 h and 24 h metagenes respectively by searching two miRNA databases. The two databases are MicroRNAdb (http://bioinfo.au.tsinghua.edu.cn/micrornadb/) and miRNAMap ( http://mirnamap.mbc.nctu.edu.tw/). Searching other databases, such as CoGemiR and miRBase, returned no information about the tissue distribution of our interested miRNAs. We found that some of our miRNAs are reported to be enriched in bone marrow (miR-142) and GI tract (miR-142, miR-150, miR-155). miR142 is highly expressed in the hemotopoietic organs such as bone marrow, spleen and thymus [36]. miR142 is also shown to have 1 clone in small intestine and 6 clones in colon. miR-150 has one clone in colon [37]. miR-155 has one clone in colon [37], [38]. The remaining miRNAs in our metagenes are either having a tissue origin from other tissues or without tissue origin information. However, as shown by Wang et al [39], the composition of circulating miRNAs might originate from diverse tissues and cell types in the body after acetaminophen-induced liver damage. It is not surprising if our miRNAs are released from tissues other than bone marrow and GI. Whether these miRNAs are released from the radiation target organs warrants further study.

Differentially expressed plasma miRNAs have been studied in many models. Plasma miR150 has been shown to be up-regulated after LPS treatment [40]; plasma miR-122 level has been shown to be increased by viral-, alcohol-, and chemical-related hepatic diseases [41]. Plasma miR208b and miR449 have been shown to be highly elevated by cardivascular damage [42]. Plasma miR-22, miR-101b, miR-122, miR -133a, miR135a*, miR-192, miR193 and miR486 have been shown to be affected by liver damage [39]. None of these differentially expressed plasma miRNAs showed up in our radiation metagenes. This suggests that our metagene is very specific for radiation injury. Our findings also strengthen the argument that certain plasma miRNAs are biomarkers for different diseases.

The mechanisms of how circulating miRNAs are released into circulation are not clear. One possibility is that cells are actively secreting vesicles or exosomes that contain miRNAs [43], [44]. Another possibility is that a ceramide dependent pathway controls the intercellular transfer of miRNAs via exosome [45]. Other research suggested that apoptotic cells release miRNAs via apoptotic body [46]. One recent study suggested that circuilating miRNAs are released by cells via Argonaut2 protein complexes [47]. A recent study also showed that released miRNAs do not necessarily reflect the abundance of miRNA in the cell of origin [48]. The releasing of miRNA to the circulation might not be a simple trash disposal mechanism [49], rather it may be a well controlled process.

Studies have shown that plasma miRNAs are stable in plasma after 24 hours' incubation at room temperature, or undergoing eight cycles of freeze-thawing [7]. The protective exsosomes, microvesicles and Argonaute2 protein complexes are responsible for the stability of plasma miRNAs [50], [51] [47]. Whether plasma miRNAs need to be dissociated from protective exsosomes, microvesicles or protein complexes before their degradation needs further studies.

In summary, our study describes a rapid screening test for the molecular dosimetry of radiation exposure. Further validation studies are required before these miRNA targets can be used for molecular biodosimetry to predict the radiation exposure. Studies are underway to validate miRNA profiling method in patients or monkeys who undergo TBI. Once validated, the miRNA expression signatures would provide an early dose estimate that would then be used when making decisions to triage patients. To our knowledge, this is the first report of use of plasma miRNA as radiation exposure biomarkers. This study provides proof-of-principle to the use of plasma miRNA as a predictive tool for radiation exposures.

## Supporting Information

Figure S1 **Detection of possible platelet contamination in plasma.** **A:** The amplification plot of platelet specific gene itga2b in the whole blood and plasma after first and second spins detected by realtime PCR. The arbitrary threshold was shown in a blue line. **B:** Platelet counts in whole blood and plasma using HemaVet machine. **C:** The fold changes of itga2b gene in whole blood, after first and second spins based on the Ct value measured in Figure S1A. **D:** The ranking in the plasma of the 10 most abundant platelet microRNAs. **E:** The ranking in the platelet of the 10 most abundant plasma microRNAs. N/A means these miRNAs were not detected in platelet.
(TIF)

## References

[1] Waselenko JK, MacVittie TJ, Blakely WF, Pesik N, Wiley AL (2004). Medical management of the acute radiation syndrome: recommendations of the Strategic National Stockpile Radiation Working Group.. Annals of Internal Medicine.

[2] Coleman CN, Stone HB, Moulder JE, Pellmar TC (2004). Medicine. Modulation of radiation injury.. Science (New York, NY).

[3] Gougelet RM, Rea ME, Nicolalde RJ, Geiling JA, Swartz HM (2010). The view from the trenches: part 1-emergency medical response plans and the need for EPR screening.. Health Phys.

[4] Blakely WF, Ossetrova NI, Whitnall MH, Sandgren DJ, Krivokrysenko VI (2010). Multiple parameter radiation injury assessment using a nonhuman primate radiation model-biodosimetry applications.. Health physics.

[5] Redon CE, Nakamura AJ, Gouliaeva K, Rahman A, Blakely WF (2010). The use of gamma-H2AX as a biodosimeter for total-body radiation exposure in non-human primates.. PLoS ONE.

[6] Chim SS, Shing TK, Hung EC, Leung TY, Lau TK (2008). Detection and characterization of placental microRNAs in maternal plasma.. Clinical chemistry.

[7] Mitchell PS, Parkin RK, Kroh EM, Fritz BR, Wyman SK (2008). Circulating microRNAs as stable blood-based markers for cancer detection.. Proceedings of the National Academy of Sciences of the United States of America.

[8] Wang J, Chen J, Chang P, LeBlanc A, Li D (2009). MicroRNAs in plasma of pancreatic ductal adenocarcinoma patients as novel blood-based biomarkers of disease.. Cancer prevention research (Philadelphia, Pa).

[9] Chiu RW, Poon LL, Lau TK, Leung TN, Wong EM (2001). Effects of blood-processing protocols on fetal and total DNA quantification in maternal plasma.. Clin Chem.

[10] Tibshirani R, Hastie T, Narasimhan B, Chu G (2002). Diagnosis of multiple cancer types by shrunken centroids of gene expression.. Proceedings of the National Academy of Sciences of the United States of America.

[11] Landry P, Plante I, Ouellet DL, Perron MP, Rousseau G (2009). Existence of a microRNA pathway in anucleate platelets.. Nat Struct Mol Biol.

[12] Greenberg ML, Chanana AD, Cronkite EP, Schiffer LM, Stryckmans PA (1968). Extracorporeal irradiation of blood in man: radiation resistance of circulating platelets.. Radiat Res.

[13] Anno GH, Young RW, Bloom RM, Mercier JR (2003). Dose response relationships for acute ionizing-radiation lethality.. Health Phys.

[14] Hall EJ (2000). Radiobiology for the radiologist.

[15] Meadows SK, Dressman HK, Daher P, Himburg H, Russell JL (2010). Diagnosis of partial body radiation exposure in mice using peripheral blood gene expression profiles.. PLoS ONE.

[16] Meadows SK, Dressman HK, Muramoto GG, Himburg H, Salter A (2008). Gene expression signatures of radiation response are specific, durable and accurate in mice and humans.. PLoS ONE.

[17] Dressman HK, Muramoto GG, Chao NJ, Meadows S, Marshall D (2007). Gene expression signatures that predict radiation exposure in mice and humans.. Plos Medicine.

[18] Muriithi EW, Belcher PR, Menys VC, Chaudhry MA, Raco L (2000). Quantitative detection of platelet aggregates in whole blood without fixation.. Platelets.

[19] Ossetrova NI, Blakely WF (2009). Multiple blood-proteins approach for early-response exposure assessment using an in vivo murine radiation model.. International journal of radiation biology.

[20] Zhang L, Zhang M, Yang S, Cao Y, Bingrong Zhang S (2010). A new biodosimetric method: branched DNA-based quantitative detection of B1 DNA in mouse plasma.. The British journal of radiology.

[21] Sharova LV, Sharov AA, Nedorezov T, Piao Y, Shaik N (2009). Database for mRNA half-life of 19 977 genes obtained by DNA microarray analysis of pluripotent and differentiating mouse embryonic stem cells.. DNA research : an international journal for rapid publication of reports on genes and genomes.

[22] Cowan ML, Vera J (2008). Proteomics: advances in biomarker discovery.. Expert Review of Proteomics.

[23] Ebert MP, Korc M, Malfertheiner P, Rocken C (2006). Advances, challenges, and limitations in serum-proteome-based cancer diagnosis.. Journal of Proteome Research.

[24] Weiner AM (2002). SINEs and LINEs: the art of biting the hand that feeds you.. Current opinion in cell biology.

[25] Li TH, Schmid CW (2001). Differential stress induction of individual Alu loci: implications for transcription and retrotransposition.. Gene.

[26] Teng SC, Kim B, Gabriel A (1996). Retrotransposon reverse-transcriptase-mediated repair of chromosomal breaks.. Nature.

[27] Chaudhry MA, Sachdeva H, Omaruddin RA (2010). Radiation-induced micro-RNA modulation in glioblastoma cells differing in DNA-repair pathways.. DNA and cell biology.

[28] Cha HJ, Shin S, Yoo H, Lee EM, Bae S (2009). Identification of ionizing radiation-responsive microRNAs in the IM9 human B lymphoblastic cell line.. International journal of oncology.

[29] Wagner-Ecker M, Schwager C, Wirkner U, Abdollahi A, Huber PE (2010). MicroRNA expression after ionizing radiation in human endothelial cells.. Radiation oncology (London, England).

[30] He L, He X, Lim LP, de Stanchina E, Xuan Z (2007). A microRNA component of the p53 tumour suppressor network.. Nature.

[31] Hermeking H (2010). The miR-34 family in cancer and apoptosis.. Cell death and differentiation.

[32] Hunter CP, Winston WM, Molodowitch C, Feinberg EH, Shih J (2006). Systemic RNAi in Caenorhabditis elegans.. Cold Spring Harbor symposia on quantitative biology.

[33] Dunoyer P, Brosnan CA, Schott G, Wang Y, Jay F (2010). An endogenous, systemic RNAi pathway in plants.. The EMBO journal.

[34] Ooi AG, Sahoo D, Adorno M, Wang Y, Weissman IL (2010). MicroRNA-125b expands hematopoietic stem cells and enriches for the lymphoid-balanced and lymphoid-biased subsets.. Proc Natl Acad Sci U S A.

[35] Gao Y, Schug J, McKenna LB, Le Lay J, Kaestner KH (2011). Tissue-specific regulation of mouse microRNA genes in endoderm-derived tissues.. Nucleic Acids Res.

[36] Chen CZ, Li L, Lodish HF, Bartel DP (2004). MicroRNAs modulate hematopoietic lineage differentiation.. Science.

[37] Lagos-Quintana M, Rauhut R, Yalcin A, Meyer J, Lendeckel W (2002). Identification of tissue-specific microRNAs from mouse.. Curr Biol.

[38] Lagos-Quintana M, Rauhut R, Meyer J, Borkhardt A, Tuschl T (2003). New microRNAs from mouse and human.. RNA.

[39] Wang K, Zhang S, Marzolf B, Troisch P, Brightman A (2009). Circulating microRNAs, potential biomarkers for drug-induced liver injury.. Proc Natl Acad Sci U S A.

[40] Vasilescu C, Rossi S, Shimizu M, Tudor S, Veronese A (2009). MicroRNA fingerprints identify miR-150 as a plasma prognostic marker in patients with sepsis.. PLoS ONE.

[41] Zhang Y, Jia Y, Zheng R, Guo Y, Wang Y (2010). Plasma microRNA-122 as a biomarker for viral-, alcohol-, and chemical-related hepatic diseases.. Clin Chem.

[42] Corsten MF, Dennert R, Jochems S, Kuznetsova T, Devaux Y (2010). Circulating MicroRNA-208b and MicroRNA-499 reflect myocardial damage in cardiovascular disease.. Circ Cardiovasc Genet.

[43] Skog J, Wurdinger T, van Rijn S, Meijer DH, Gainche L (2008). Glioblastoma microvesicles transport RNA and proteins that promote tumour growth and provide diagnostic biomarkers.. Nat Cell Biol.

[44] Hunter MP, Ismail N, Zhang X, Aguda BD, Lee EJ (2008). Detection of microRNA expression in human peripheral blood microvesicles.. PLoS ONE.

[45] Kosaka N, Iguchi H, Yoshioka Y, Takeshita F, Matsuki Y (2010). Secretory mechanisms and intercellular transfer of microRNAs in living cells.. J Biol Chem.

[46] Zernecke A, Bidzhekov K, Noels H, Shagdarsuren E, Gan L (2009). Delivery of microRNA-126 by apoptotic bodies induces CXCL12-dependent vascular protection.. Sci Signal.

[47] Arroyo JD, Chevillet JR, Kroh EM, Ruf IK, Pritchard CC (2011). Argonaute2 complexes carry a population of circulating microRNAs independent of vesicles in human plasma.. Proc Natl Acad Sci U S A.

[48] Pigati L, Yaddanapudi SC, Iyengar R, Kim DJ, Hearn SA (2010). Selective release of microRNA species from normal and malignant mammary epithelial cells.. PLoS ONE.

[49] Johnstone RM, Mathew A, Mason AB, Teng K (1991). Exosome formation during maturation of mammalian and avian reticulocytes: evidence that exosome release is a major route for externalization of obsolete membrane proteins.. J Cell Physiol.

[50] Fevrier B, Raposo G (2004). Exosomes: endosomal-derived vesicles shipping extracellular messages.. Curr Opin Cell Biol.

[51] Heijnen HF, Schiel AE, Fijnheer R, Geuze HJ, Sixma JJ (1999). Activated platelets release two types of membrane vesicles: microvesicles by surface shedding and exosomes derived from exocytosis of multivesicular bodies and alpha-granules.. Blood.

