# Supplementary figures and images for: Plasma miRNA as Biomarkers for Assessment of Total-Body Radiation Exposure Dosimetry
Source: PLoS One. 2011 Aug 17;6(8):e22988. doi: 10.1371/journal.pone.0022988 (PMC3157373; doi:10.1371/journal.pone.0022988)

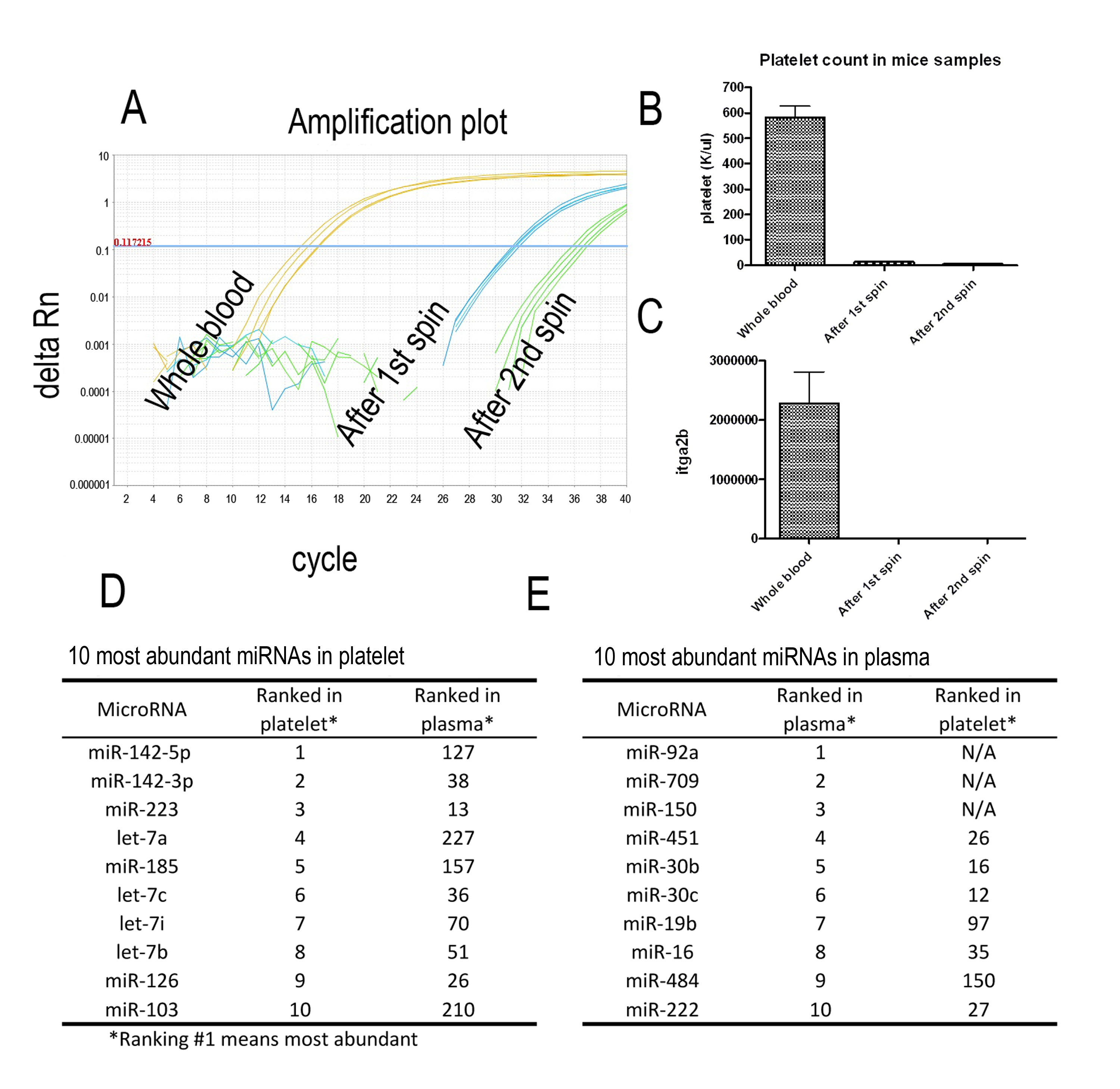

Supplement: Figure S1 — Detection of possible platelet contamination in plasma. A: The amplification plot of platelet specific gene itga2b in the whole blood and plasma after first and second spins detected by realtime PCR. The arbitrary threshold was shown in a blue line. B: Platelet counts in whole blood and plasma using HemaVet machine. C: The fold changes of itga2b gene in whole blood, after first and second spins based on the Ct value measured in Figure S1A. D: The ranking in the plasma of the 10 most abundant platelet microRNAs. E: The ranking in the platelet of the 10 most abundant plasma microRNAs. N/A means these miRNAs were not detected in platelet. (TIF) [file pone.0022988.s001.tif]
